# Differential contribution of *Anopheles coustani* and *Anopheles arabiensis* to the transmission of *Plasmodium falciparum* and *Plasmodium vivax* in two neighbouring villages of Madagascar

**DOI:** 10.1186/s13071-020-04282-0

**Published:** 2020-08-26

**Authors:** Jessy Goupeyou-Youmsi, Tsiriniaina Rakotondranaivo, Nicolas Puchot, Ingrid Peterson, Romain Girod, Inès Vigan-Womas, Richard Paul, Mamadou Ousmane Ndiath, Catherine Bourgouin

**Affiliations:** 1 Immunology of Infectious Diseases Unit, Institut Pasteur de Madagascar, Antananarivo, Madagascar; 2 Functional Genetics of Infectious Diseases Unit, Institut Pasteur, Paris, France; 3 Doctoral School “Complexité du Vivant”, Sorbonne University, Paris, France; 4 G4 Malaria Group, Institut Pasteur de Madagascar, Antananarivo, Madagascar; 5 Doctoral School “Génie du vivant et modélisation” Mahajanga University, Mahajanga, Madagascar; 6 Centre National de la Recherche Scientifique UMR2000, Institut Pasteur, Paris, France; 7 Center for Vaccine Development and Global Health, University of Maryland School of Medicine, Baltimore, Maryland USA; 8 Medical Entomology Unit, Institut Pasteur de Madagascar, Antananarivo, Madagascar

**Keywords:** *Anopheles coustani*, *Anopheles arabiensis*, *Plasmodium falciparum*, *Plasmodium vivax*, Vector biology dynamics, Andriba, Madagascar

## Abstract

**Background:**

Malaria is still a heavy public health concern in Madagascar. Few studies combining parasitology and entomology have been conducted despite the need for accurate information to design effective vector control measures. In a Malagasy region of moderate to intense transmission of both *Plasmodium falciparum* and *P. vivax*, parasitology and entomology have been combined to survey malaria transmission in two nearby villages.

**Methods:**

Community-based surveys were conducted in the villages of Ambohitromby and Miarinarivo at three time points (T1, T2 and T3) during a single malaria transmission season. Human malaria prevalence was determined by rapid diagnostic tests (RDTs), microscopy and real-time PCR. Mosquitoes were collected by human landing catches and pyrethrum spray catches and the presence of *Plasmodium* sporozoites was assessed by TaqMan assay.

**Results:**

Malaria prevalence was not significantly different between villages, with an average of 8.0% by RDT, 4.8% by microscopy and 11.9% by PCR. This was mainly due to *P. falciparum* and to a lesser extent to *P. vivax*. However, there was a significantly higher prevalence rate as determined by PCR at T2 ($\chi_{2}^{2}$ = 7.46, *P* = 0.025). Likewise, mosquitoes were significantly more abundant at T2 ($\chi_{2}^{2}$ = 64.8, *P* < 0.001), especially in Ambohitromby. At T1 and T3 mosquito abundance was higher in Miarinarivo than in Ambohitromby ($\chi_{2}^{2}$ = 14.92, *P* < 0.001). Of 1550 *Anopheles* mosquitoes tested, 28 (1.8%) were found carrying *Plasmodium* sporozoites. The entomological inoculation rate revealed that *Anopheles coustani* played a major contribution in malaria transmission in Miarinarivo, being responsible of 61.2 infective bites per human (ib/h) during the whole six months of the survey, whereas, it was *An. arabiensis*, with 36 ib/h, that played that role in Ambohitromby.

**Conclusions:**

Despite a similar malaria prevalence in two nearby villages, the entomological survey showed a different contribution of *An. coustani* and *An. arabiensis* to malaria transmission in each village. Importantly, the suspected secondary malaria vector *An. coustani*, was found playing the major role in malaria transmission in one village. This highlights the importance of combining parasitology and entomology surveys for better targeting local malaria vectors. Such study should contribute to the malaria pre-elimination goal established under the 2018–2022 National Malaria Strategic Plan. 
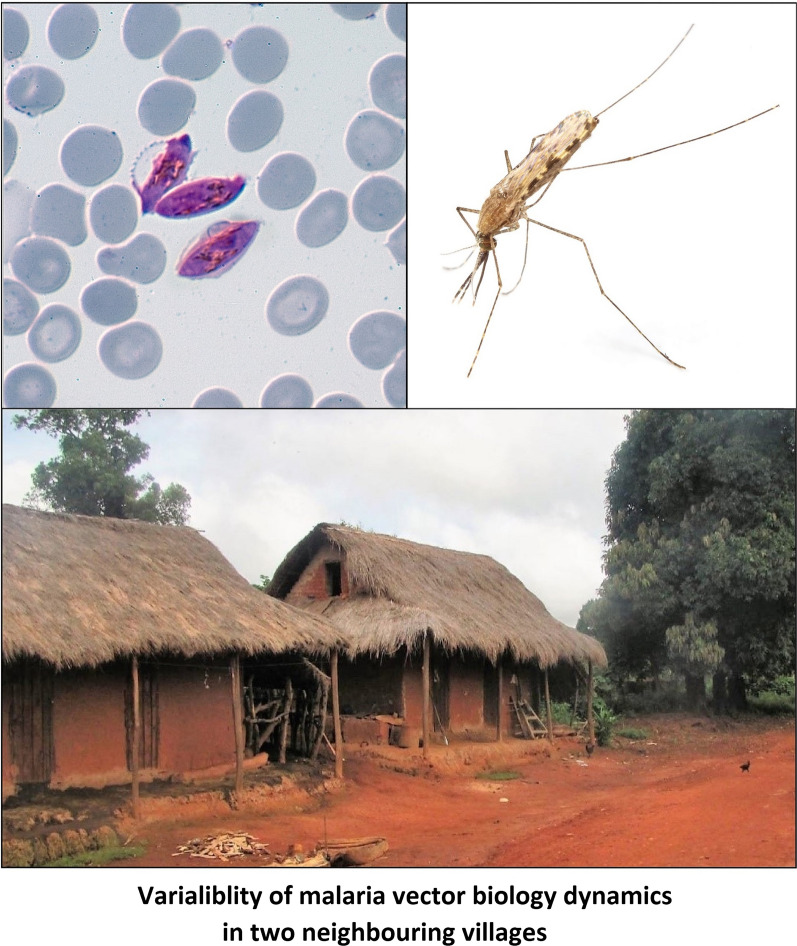

## Background

Malaria remains a major public health concern in Madagascar, with an increase in the number of cases and deaths in years 2017 and 2018, compared to the year 2016 [1]. Malaria epidemiology in Madagascar is highly heterogenous and varies according to the climatic and ecological environment that allows a stratification in bioclimatic zones and ecozones [2, 3]. All four human malaria species are circulating, with *Plasmodium falciparum* being the most prevalent. Across the ecozones, the average prevalence of *P. falciparum* varies from 2 to 12% [3] but can reach 30% in some areas [4]. Among the 26 *Anopheles* species described in the country, six have been reported as malaria vectors with different role according to geography and behaviour [5]. Three species belong to the *Anopheles gambiae* complex: *Anopheles gambiae* (*sensu stricto*), *An. arabiensis* and *An. merus*, the latter having a minor role in malaria transmission and being restricted to the most southern region of Madagascar [6]. Of the two other members of the *An. gambiae* complex, *An. arabiensis* is prevalent throughout Madagascar and plays a major role in malaria transmission along with *An. funestus* [7]. *Anopheles mascarensis*, endemic to Madagascar, and *An. coustani*, act as local or minor vectors [8–10].

Recent surveys of malaria incidence and prevalence between 2010–2016 confirm the heavy malaria burden for the population living in the western part of Madagascar [3, 4, 11]. In the Tsiroanomandidy district which constitutes a bridge area between the low transmission Central Highlands and the high endemic western region, both *P. falciparum* and *P. vivax* circulate. In this area, malaria prevalence and *Anopheles* species distribution have been described in detail [7, 12, 13]. Such combined parasitological and entomological information is critical for developing effective strategies for interrupting malaria transmission and moving towards malaria elimination which has been set up on the agenda of the 2018–2022 Malagasy Malaria Strategic Plan as geographically progressive elimination. Toward this goal, is reported here a combined parasitological and entomological survey in the Maevatanana district, located in the northwestern ecozone of Madagascar, and which faces a high malaria burden due to both *P. falciparum* and *P. vivax*. The study was conducted in two neighbouring villages in Andriba, a rural commune located at the transition between the western fringe of the Central Highlands (low malaria prevalence) and the northwestern ecozone (moderate to high prevalence) according to Howes et al. [3]. The main goal of this study was to determine which *Anopheles* species contribute to the local malaria transmission with the aim for providing targeted vector control recommendation to the local authorities. To our knowledge, no such combined parasitological and entomological survey has ever been performed in that region.

## Methods

### Study design and setting

**Fig. 1:**
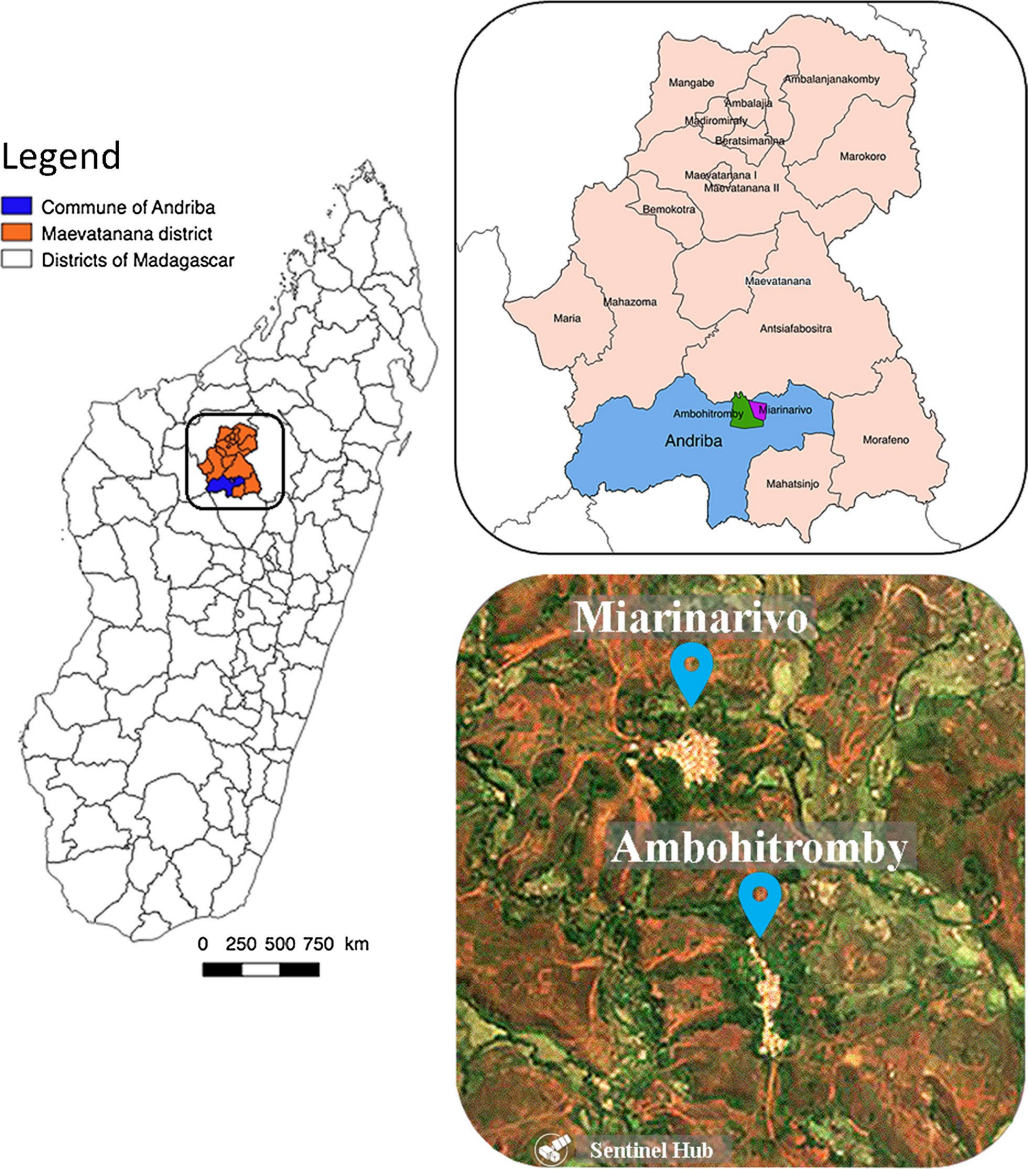
Study site. The map of Madagascar is depicted in the left panel with a focus on the Andriba region presented in more details in the upper right panel. The bottom right panel is a satellite image of the study villages, Ambohitromby and Miarinarivo (Modified Copernicus Sentinel data [2019]/Sentinel Hub)

**Fig. 2:**
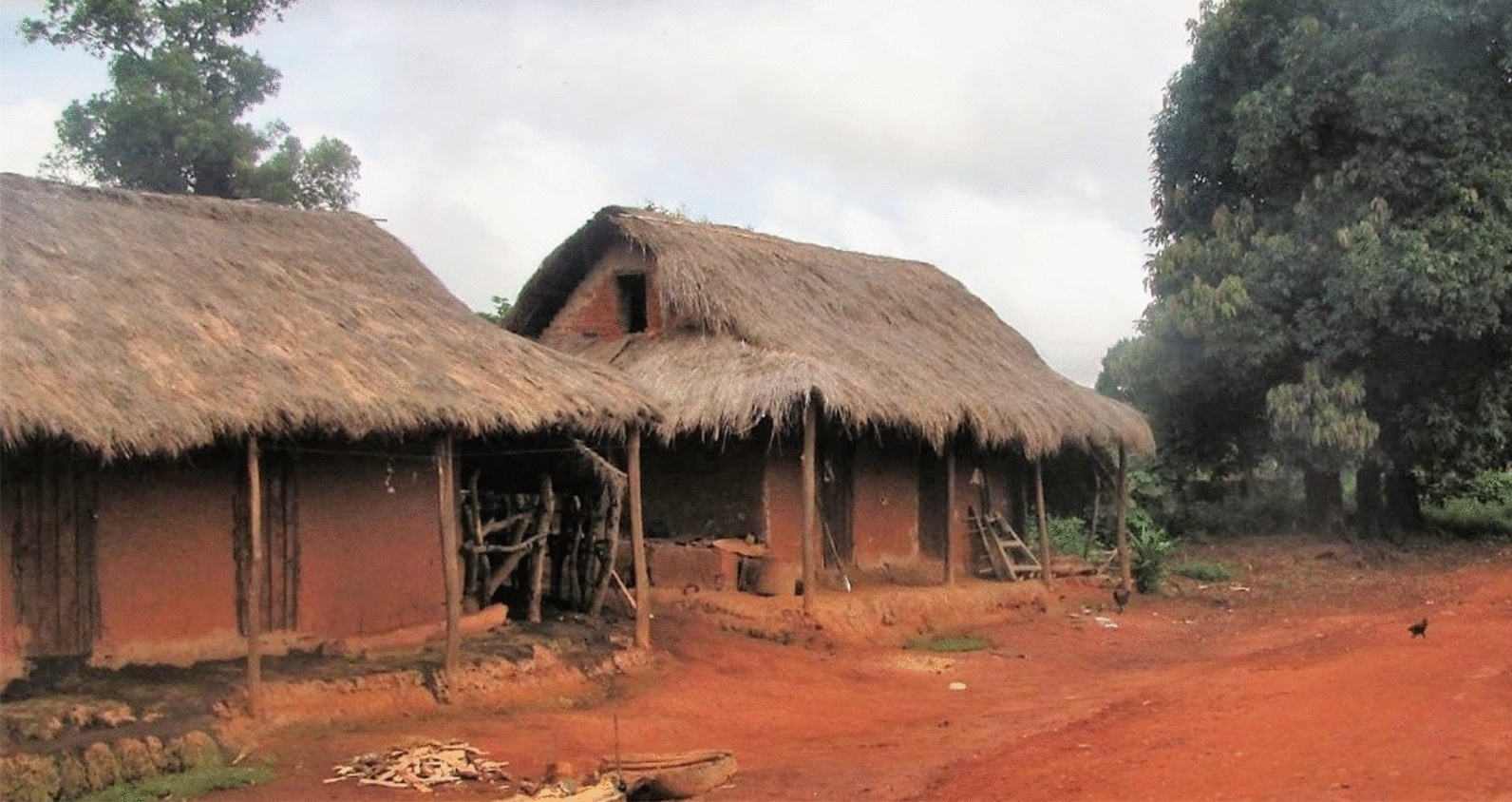
Typical Malagasy houses in Andriba rural area. The houses are built with adobe walls and thatched roofs, and usually composed of one or two rooms. The picture was taken in the village of Ambohitromby located in the rural commune of Andriba, Madagascar

The study was conducted in two villages of the rural commune of Andriba (Maevatanana district, Madagascar) which is located in the tropical northwest region of Madagascar. Andriba is characterised by a dry season that generally lasts from April to October and a rainy season from November to April; the average annual temperature is 24 °C and the average annual rainfall is 1828 mm [14]. The two villages, Ambohitromby (17°34′23.7″S, 46°55′21.4″E) and Miarinarivo (17°33′56.7″S, 46°55′10.8″E) are located 1.5 km apart and 6 km from Andriba town hall (Fig. 1), with a population of 384 and 302 inhabitants, respectively. The houses in the two villages were of typical Malagasy construction common in the rural areas of the Maevatanana district: thatched roofs, adobe walls and composed of 1 to 2 rooms (Fig. 2). Parasitological and entomological data were collected at three time points during a single malaria transmission season: at the onset of the season (November and December 2016, labelled T1); mid-season (February 2017, labelled T2); and late-season (April and May 2017, labelled T3). The latter time point was selected to correspond with the cessation of malaria transmission, but this depends upon annual climatic variation. Additional parasitological data were recorded in March 2016 and in March 2018 as part of active malaria parasite surveillance in school-age children (Bourgouin et al. unpublished data).

### Malaria prevalence in the human population

#### Blood sample collection

Study participants included village residents among whom volunteers were involved in human landing catches (HLCs). The week before each survey, a community sensitisation was organized with the help of the staff of the Andriba health centre and the presence of the head of the village explaining the benefit of malaria parasite detection. On the day of the survey, people gathered at a central point proposed by the head of the village, usually the local school. Each participant was provided a short questionnaire to collect name, age, sex, and any health conditions that might exclude them from the survey (such as dizziness or heavy treatment; neither of them was encountered). Blood samples were collected from both children and adults without clinical signs of malaria at the time of the survey (asymptomatic individuals). According to the study protocol, any individual who would have come with malaria symptoms would have been referred to the Andriba health centre. We did not encounter such a situation. Temperature and weight where recorded for each participant showing a positive rapid diagnostic test (RDT) for malaria. Participant’s names were used for longitudinal follow-up of their participation at the next surveys, but names were encoded for data analysis.

Blood samples were obtained by finger prick to perform RDTs (SD Bioline Malaria Ag P.f/Pan; Standard Diagnostics Inc., Suwon City, South Korea), thick and thin blood smears, and blood spots on filter paper (standard Whatman 3MM filter paper). The Bioline RDT enabled specific detection of *P. falciparum*, and any of *P. vivax*, *P. ovale* and *P. malariae*, as all four species are present in Madagascar. *Plasmodium* species, parasite stages and parasite density were further determined by microscopic observation of the blood smears stained with 10% Giemsa, using a light microscope (100×). A thin blood smear slide was declared malaria-negative when *Plasmodium* parasites were not detected after examination of 100 high power microscopic fields. Slides were read for asexual parasites and gametocytes, enumerated against 500 leucocytes and expressed as density/μl assuming an average leucocyte count of 8,000/μl of blood (data not shown). Individuals with positive RDT were treated with artemisinin-based combination therapy (ACT), according to national guidelines.

#### Detection of Plasmodium parasites by PCR using dried blood spots

Dried blood spots (DBS) were lysed overnight at 4 °C in 150 μl per microtube of 1× HBS buffer (Hepes buffered saline) supplemented with 0.5% saponin, final concentration. Samples were then washed twice with 1× PBS and DNA extracted with Instagene® Matrix resin (Bio-Rad Laboratories Inc., Hercules, California, USA) according to manufacturer’s instructions. Molecular detection and species identification of *Plasmodium* parasites were performed in two steps as previously described by Canier et al. [15]. *Plasmodium* spp. were first detected by a real-time PCR using genus-specific primers targeting the *Plasmodium* cytochrome *b* gene. Then, *Plasmodium* species identification was performed on DNA samples identified as positive for *Plasmodium* using a nested real-time PCR assay [15].

### Entomological data

#### Mosquito collection

Mosquitoes were collected by HLCs and indoor pyrethrum spray catches (PSCs), following WHO protocols [16]. For both HLCs and PSCs, houses were chosen randomly at the first time point, and then according to the availability of the houses. Therefore, some houses were sampled with repetition. For each of the three surveys throughout the malaria season, adult volunteers performed HLCs from 18:00 h to 06:00 h in 2 houses per night, for 3 consecutive nights. For each house, one volunteer sat inside and another outside at more than 15 m from the house; capture stations in the village were distributed ensuring a distance of more than 15 m between volunteers. The indoor and outdoor volunteers changed places every hour to minimise individual bias. The entire night was worked in two shifts, with two volunteers working between 18:00 h and 24:00 h, and a second set of volunteers from 24:00 h to 06:00 h. Thus, for each sampling period, 12 human-nights (HNs) of data were collected from each village, with a total of 72 HNs for the entire study.

PSCs were conducted in 5 houses/day/village, choosing houses that were not used for HLCs and in which no insecticide or repellent had been used during the previous week. Some of the houses were using insecticide-treated bed nets. The GPS (global positioning system) coordinates of each house was recorded as well as the date at which the PSC was performed. PSCs were done on 3 consecutive days from 06:00 h to 08:00 h each morning following the HLCs. No insecticide residual spraying had occurred in the villages from 2016 till 2018. PSCs were performed using a pyrethroid mixture of prallethrin, tetramethrin, and deltamethrin. Spraying was performed from outside of the houses, into openings, holes in walls and eaves, then in the rooms, following WHO procedures. Knockdown mosquitoes were then collected by hand picking.

#### Mosquito species identification

All mosquitoes were identified morphologically using the determination keys of Grjebine [17] and De Meillon [18]. To discriminate *An. gambiae* from *An. arabiensis*, a TaqMan assay was used targeting the intergenic spacer region of rDNA as described by Walker et al. [19], following the initial work of Scott et al. [20]. Primers and sequences used are listed in Additional file 1: Table S1. PCR reactions (20 μl) contained 5 μl of genomic DNA (see extraction procedure below), 4 μl of 5× HOT FIREPol® Probe qPCR Mix Plus/no ROX (Solis Biodyne, Tartu, Estonia), 300 nM of each primer and 200 nM of each probe. Reactions were run on a StepOnePlus (Applied Biosystems, Waltham, Massachusetts, USA) using the following temperatures: an initial step at 95 °C for 10 min, followed by 40 cycles of denaturation at 95 °C for 20 s and annealing/elongation at 60 °C for 1 min.

### *Plasmodium* detection in *Anopheles* mosquitoes

#### DNA extraction and quality control

Genomic DNA from *Anopheles* head-thorax was extracted using the DNAzol® Reagent (Thermo Fisher Scientific, Waltham, Massachusetts, USA). Briefly, the head-thorax from each mosquito was put individually in a tube; care was taken to rinse the dissecting equipment in 70% ethanol between each mosquito. A volume of 150 µl of DNAzol was added and mosquito tissues were crushed using an individual conical plastic pestle. DNA was then extracted following the manufacturer’s protocol. After precipitation, the DNA pellet was suspended in a final volume of 50 µl of nuclease-free water. DNA quality was controlled using a SYBR Green real-time PCR assay targeting the ribosomal S7 protein encoding gene. This gene is highly conserved among species belonging to the same genus. Primers previously designed against the *An. gambiae* S7 gene [21] were aligned against all available *Anopheles* S7 sequences to ensure that those primers will efficiently amplify the S7 gene fragment from any *Anopheles* captured in the field. Amplification conditions were validated on a subset of laboratory and field-collected mosquito samples including species of the subgenera *Anopheles*, *Cellia* and *Nyssorhynchus* (not shown). Amplification using the PowerSYBER® Green Master mix (Applied Biosystems) was performed as follows: an initial step at 95 °C for 15 min; followed by 40 cycles of denaturation at 95 °C for 45 s, annealing at 55 °C for 30 s and elongation at 60 °C for 45 s. Specificity of the amplification was assessed by viewing the melting curves.

#### Plasmodium detection

The detection of human *Plasmodium* gDNA in mosquitoes was performed in 2 steps. The first step used a TaqMan PCR assay targeting a region of the *18S* rRNA gene conserved among the human infecting *Plasmodium* species. For this assay, primers and probe previously described [22] have been used, with a MGB probe as in Taylor et al. [23]; this combination was previously validated for *Plasmodium* detection in mosquitoes [24]. The *Plasmodium* TaqMan probe was labelled with 5’ NED. PCR reactions (20 μl) contained 5 μl of mosquito genomic DNA, 4 μl of 5× HOT FIREPol® Probe qPCR Mix Plus/no ROX (Solis Biodyne), 300 nM of each primer and 200 nM of probe. Reactions were run on a StepOnePlus (Applied Biosystems) using the following conditions: an initial step at 95 °C for 10 min; followed by 50 cycles of denaturation at 95 °C for 20 s and annealing/elongation at 60 °C for 1 min. *Plasmodium falciparum* genomic DNA extracted from NF54 parasite cultures was used as a positive control. Any sample with amplification signal before the 38th cycle was considered positive. For high throughput screening, a pool strategy was used [25]; equal volumes of genomic DNA (extracted as described above) from 6 mosquitoes of the same species were pooled. The *Plasmodium* TaqMan assay was run using 5 µl of each DNA pool in triplicate. Mosquitoes from positive pools were then analysed individually using the same protocol as for pools. All samples positive in the TaqMan assay were then analysed for the identification of *P. falciparum* and *P. vivax* species. For each positive sample, 2 distinct real-time SYBR Green PCR assays were done using species-specific primers targeting the *cytochrome b* gene. Purified gDNA from *P. falciparum* and *P. vivax* were used as positive controls. Each reaction was run in triplicate. The real-time PCR conditions were as previously described [15]: an initial step at 95 °C for 15 min; followed by 40 cycles of denaturation at 95 °C for 20 s and annealing/elongation at 60 °C for 1 min. Sequences of the primers and TaqMan probes used for the *Plasmodium* detection in *Anopheles* mosquitoes are listed in Additional file 1: Table S1.

### Statistical analysis

*Plasmodium* spp. prevalence rates, as determined by microscopy, RDT and PCR were analysed by fitting a generalized linear mixed model (GLMM) with binomial error structure (i.e. a logistic regression) with individual as the random factor and village, time point (and their interaction), age and sex as explanatory variables. Total mosquito numbers from HLC were analysed by fitting a GLMM with Poisson error structure (i.e. a loglinear regression) with house identification number as the random factor and village, time point (and their interaction) as explanatory variables. For endophagy, mosquito species-specific analyses were similarly performed individually for all mosquito species for which more than a grand total of 50 individual mosquitoes were collected. In addition to village and time point (and their interaction), place of capture (indoors *vs* outdoors) was also included in the model. All GLMM analyses were performed in GenStat version 15 (GenStat for Windows 15th Edition, VSN International Ltd., Hemel Hempstead, UK.). To account for any underdispersion or overdispersion in the data, a dispersion heterogeneity was used in the analyses.

## Results

### *Plasmodium* carriage in the human population

The parasitological survey involved 380 individuals (218 in Ambohitromby and 162 in Miarinarivo, fairly representative of more than 50% of the population, ranging from 5 months to 68 years-old (Additional file 1: Table S2). No participant with clinical signs of malaria was encountered during the surveys. A total of 590 samples (351 in Ambohitromby and 239 in Miarinarivo) were analysed by RDT, microscopy and real-time PCR. Human malaria prevalence was 8.0% by RDT, 4.8% by microscopy and 11.9% by real-time PCR over the whole study (Table 1). There were no significant associations for any variables for *Plasmodium* spp. prevalence rates when determined by microscopy or RDT. However, there was a significantly higher prevalence rate as determined by PCR at T2 ($\chi_{2}^{2}$ = 7.46, *P* = 0.025). There were no differences between villages, nor by age or sex. Except for T3 in Miarinarivo, the PCR technique, as expected, was able to detect a greater number of parasite carriers over RDT and microscopy, revealing a substantial proportion of sub-microscopic parasite carriers. The lower proportion of PCR-positive samples at T3 in Miarinarivo, compared to T1 and T2, might result from inadequate conservation of the blood spots (12 of them) before PCR processing.

**Table 1:** Prevalence of *Plasmodium* infections in asymptomatic individuals assessed by RDT, microscopy and real-time PCR

		RDT				Microscopy				Real-time PCR			
		T1	T2	T3	Total	T1	T2	T3	Total	T1	T2	T3	Total
Ambohitromby	*n*	173	79	99	351	173	79	99	351	173	79	99	351
	Positive	13	10	5	28	9	3	3	15	20	12	10	42
	Prevalence (%)	7.5	12.7	5.1	8.0	5.2	3.8	3.0	4.3	11.6	15.2	10.1	12.0
Miarinarivo	*n*	122	65	52	239	122	65	49	236	122	65	52^a^	239
	Positive	9	7	3	19	7	3	3	13	14	12	2	28
	Prevalence (%)	7.4	10.8	5.8	7.9	5.7	4.6	6.1	5.5	11.5	18.5	3.8	11.7
Prevalence of *Plasmodium* infections in the two villages		7.5	11.8	5.3	8.0	5.4	4.2	4.1	4.8	11.5	16.7	7.9	11.9

^a^12 blood spots on filter paper have not been properly preserved. The same individuals were involved during the transversal parasitological study including the three methods of malaria diagnostic

*Notes*: In Ambohitromby, a total of 173, 79 and 99 samples were analysed for T1, T2 and T3 respectively. In Miarinarivo, a total of 122, 65 and 52 samples were analysed for T1, T2 and T3, respectively, except at T3 were only 49 smears were read as 3 slides were unreadable

*Abbreviations*: n, sample size

Among the 70 positive samples identified by real-time PCR, 84.3% carried *P. falciparum*, 5.7% carried *P. vivax*, 1.4% carried *P. malariae* and 8.6% carried mixed infections always involving *P. falciparum* (Table 2). All mixed infections were observed in Ambohitromby.

**Table 2:** *Plasmodium* species detected by real-time PCR in asymptomatic individuals at the three time points (T1-T3)

	Ambohitromby				Miarinarivo				Total by species (%)
	T1	T2	T3	Total	T1	T2	T3	Total	
Sample size	173	79	99	351	122	65	52	239	590
*P. falciparum*	17	9	7	33	13	11	2	26	59 (84.3)
*P. vivax*	1	1	1	3	0	1	0	1	4 (5.7)
*P. malariae*	0	0	0	0	1	0	0	1	1 (1.4)
Mixed infection^a^	2	2	2	6	0	0	0	0	6 (8.6)
Total by village and time point (%)	20 (11.6)	12 (15.2)	10 (10.1)	42 (12.0)	14 (11.5)	12 (18.5)	2 (3.8)	28 (11.7)	70 (100)

^a^The 6 mixed infections implicate *P. falciparum* with *P. vivax* (4), *P. malariae* (1) and *P. ovale* (1)

### Mosquito species and behaviour

#### Abundance and diversity

In total, 2407 mosquitoes were collected during 72 HNs in Ambohitromby and Miarinarivo. As presented in Table 3, *Anopheles* was the most abundant mosquito genus collected (68.55%, *n* = 1650) followed by *Culex* (26.87%, *n* = 647), *Mansonia* (3.49%, *n* = 84), *Aedes* (0.99%, *n* = 24) and *Coquillettidia* (0.08%, *n* = 2). Among *Anopheles*, *An. coustani* was by far the most abundant representing 52.99% (751/1417) of the known potential malaria vectors in Madagascar, followed by *An. arabiensis* (28.93%, 410/1417), *An. funestus* (12.84%, 182/1417) and *An. mascarensis* (4.66%, 66/1417); *An. gambiae* was barely represented (0.56%, 8/1417). Detailed data covering all collected species are presented in Additional file 1: Table S3.

**Table 3:** Mosquitoes collected by HLCs in Ambohitromby and Miarinarivo at the three time points T1-T3)

Mosquito species	Ambohitromby				Miarinarivo				Total by species	Proportion (in %)
	T1	T2	T3	Total	T1	T2	T3	Total		
*Anopheles coustani* ^a^	75	42	162	279	66	97	309	472	751	31.20
*Anopheles arabiensis* ^a,b^	64	207	10	281	16	81	32	129	410	17.03
*Anopheles funestus* ^a^	34	26	40	100	9	14	59	82	182	7.56
*Anopheles squamosus/cydippis*	13	43	29	85	8	40	15	63	148	6.15
*Anopheles mascarensis* ^a^	24	13	12	49	5	2	10	17	66	2.74
*Anopheles rufipes*	9	10	0	19	5	11	9	25	44	1.83
*Anopheles maculipalpis*	7	9	0	16	10	9	5	24	40	1.66
*Anopheles gambiae* ^a,b^	0	4	0	4	1	2	1	4	8	0.33
*Anopheles pretoriensis*	0	0	0	0	0	0	1	1	1	0.04
Total *Anopheles*	226	354	253	833	120	256	441	817	1650	68.55
*Culex antennatus*	30	258	4	292	49	99	25	173	465	19.32
*Culex quinquefasciatus*	5	103	0	108	15	23	19	57	165	6.86
*Culex giganteus*	0	0	0	0	2	4	2	8	8	0.33
*Culex univittatus*	4	0	0	4	0	0	0	0	4	0.17
*Culex decens*	1	0	0	1	2	0	0	2	3	0.12
*Culex bitaeniorhyncus*	0	0	0	0	1	1	0	2	2	0.08
Total *Culex*	40	361	4	405	69	127	46	242	647	26.88
*Mansonia uniformis*	8	9	3	20	5	25	34	64	84	3.49
Total *Mansonia*	8	9	3	20	5	25	34	64	84	3.49
*Aedes tiptoni*	1	0	0	1	8	0	1	9	10	0.42
*Aedes skusea*	5	0	0	5	3	0	0	3	8	0.33
*Aedes albopictus*	0	1	0	1	1	1	0	2	3	0.12
*Aedes vittatus*	1	1	0	2	0	0	0	0	2	0.08
*Aedes circumlateolus*	0	0	0	0	0	0	1	1	1	0.04
Total *Aedes*	7	2	0	9	12	1	2	15	24	1.00
*Coquillettidia grandidieri*	0	0	0	0	0	0	2	2	2	0.08
Total *Coquillettidia*	0	0	0	0	0	0	2	2	2	0.08
Total by village and time point	281	726	260	1267	206	409	525	1140	2407	_

^a^Known potential malaria vectors in Madagascar

^b^*An. arabiensis* and *An. gambiae* were identified by TaqMan assay among all *An. gambiae* (*s.l*.) collected (see Methods section)

*Note*: The proportion is equal to the total by species divided by the total of all species collected (*n* = 2407)

**Fig. 3:**
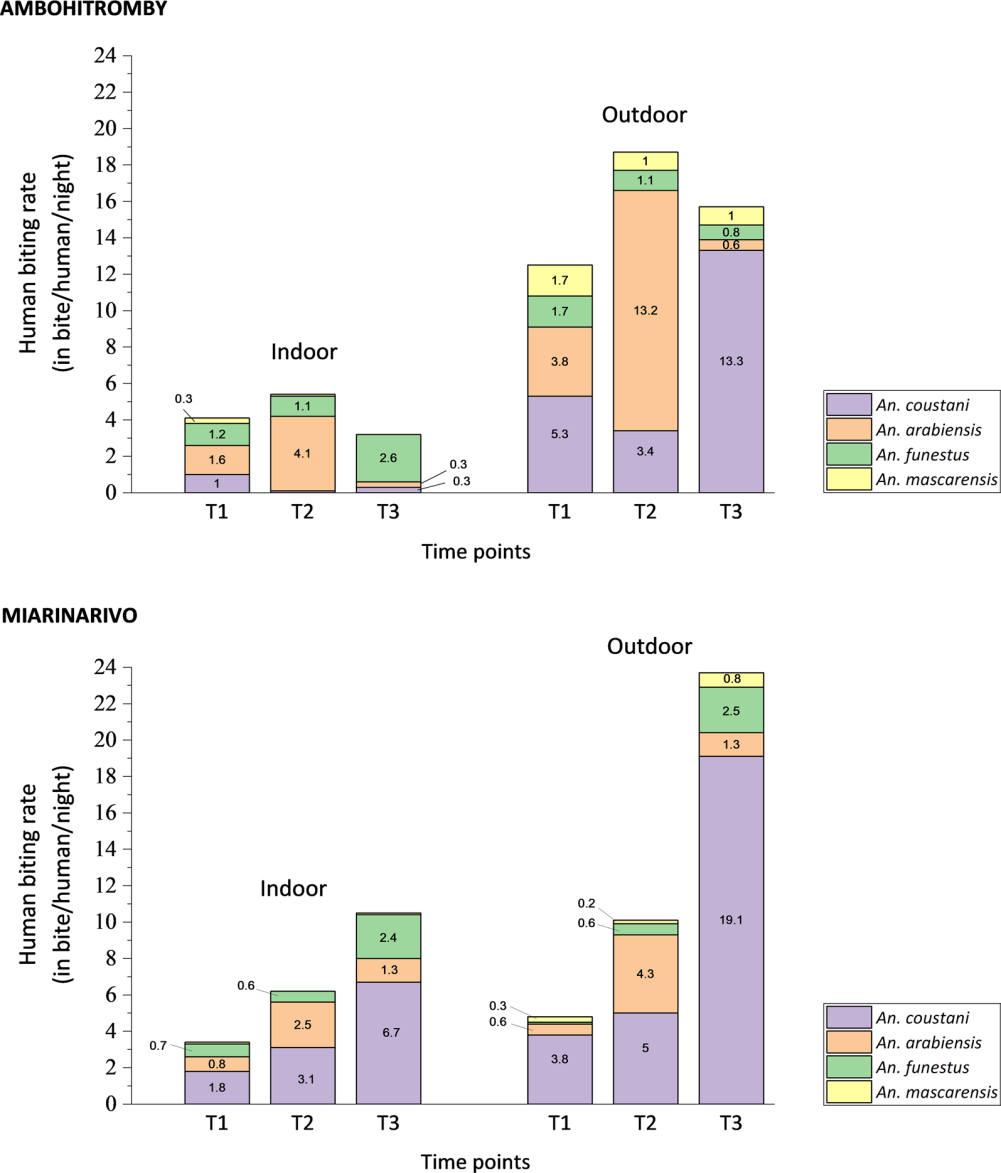
Indoor and outdoor human-biting rate of malaria vectors at the three time points in Ambohitromby and Miarinarivo. Light numbers within the graphs indicate the mean bite per human and per night for each of the four *Anopheles* species

Analysis of total *Anopheles* caught by HLC revealed significant associations with time point and an interaction with village. The number of *Anopheles* was higher at T2 ($\chi_{2}^{2}$ = 64.8, *P* < 0.001), especially in Ambohitromby, whereas at T1 and T3, numbers were higher in Miarinarivo (interaction term $\chi_{2}^{2}$ = 14.92, *P* < 0.001). The human-biting rate (HBR), corresponding to the number of collected mosquitoes per number of human-nights, was determined for the known potential malaria vectors, but *An. gambiae* due to the low number of captured mosquitoes (*n* = 8). Results over time and for each village are depicted in Fig. 3. Overall *Anopheles* HBR varies in each village over the time course of the survey, from 8 bites per human and per night (b/h/n) to 34.3 b/h/n. However, the bite frequency was higher at T2 in Ambohitromby, while being the highest at T3 in Miarinarivo. Strikingly, *An. arabiensis* was the malaria vector species with the highest HBR in Ambohitromby, with 17.3 b/h/n at T2, while in Miarinarivo, it was *An. coustani* with 25.9 b/h/n at T3. Nevertheless, *An. coustani* exhibited the highest biting frequency in both villages at T3, which corresponds to the time where the rice reached full maturity providing high shade on the padding fields suitable for *An. coustani* larval development.

**Fig. 4:**
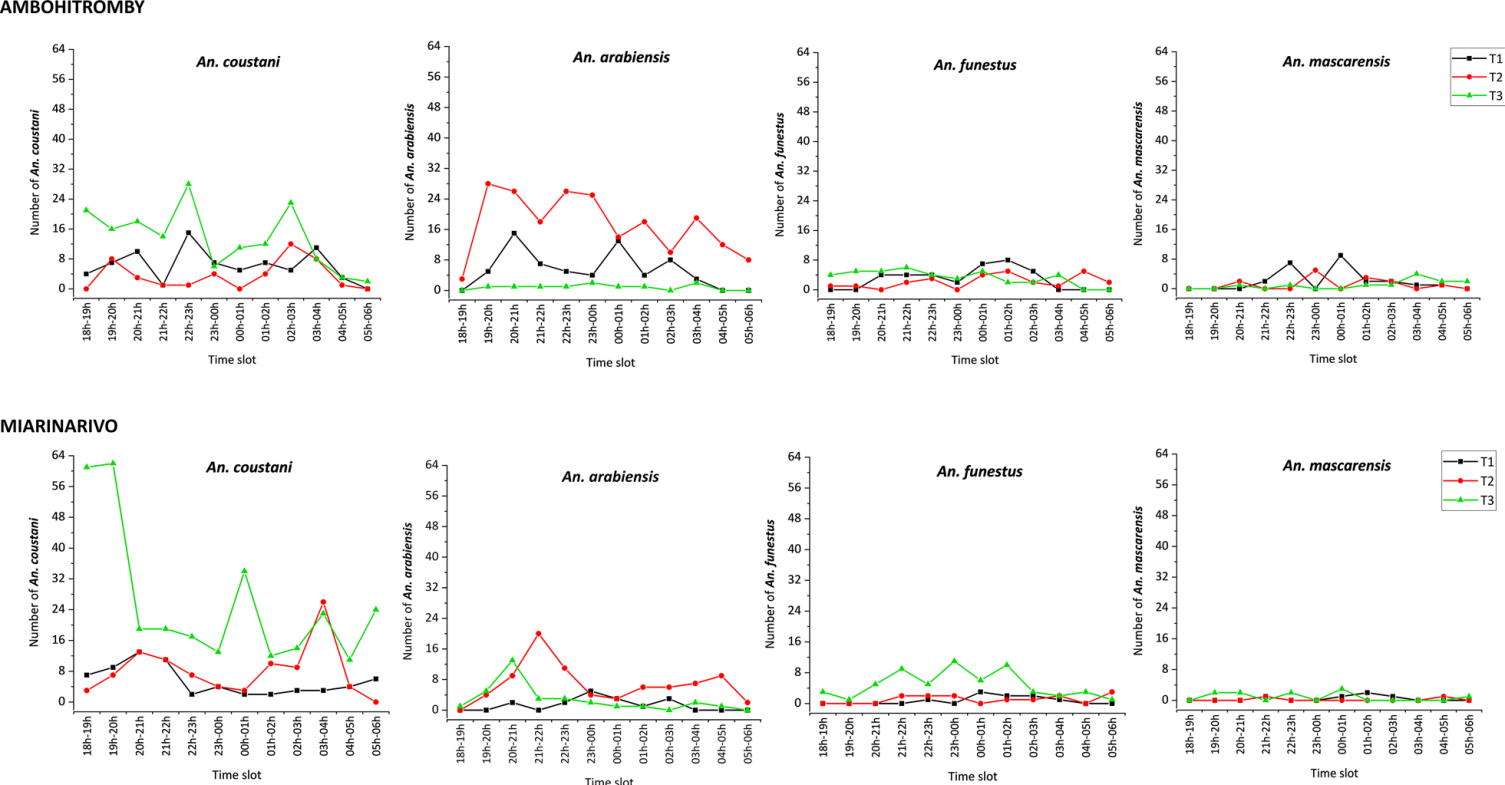
Hourly biting rate of malaria vectors at the three time points in Ambohitromby and Miarinarivo. Data represent both indoor and outdoor HLCs collected mosquitoes

Looking at the hourly biting rate of the four potential malaria vectors across time-points in the two villages, the four mosquito species mostly bit all night long (Fig. 4). There were however variations in the biting pattern across the night and according to the time point, especially for *An. coustani* and *An. arabiensis*. Considering *An. coustani*, the HBR in the two villages was higher at T3 compared to T1 and T2. The highest HBR peak at T1 was between 22:00–23:00 h in Ambohitromby and between18:00–20:00 h in Miarinarivo. The HBR peak at T2 was higher in Miarinarivo compared to Ambohitromby and occurred between 03:00–04:00 h. At T3, the biting pattern over the night was overall similar between the two villages. The *An. arabiensis* HBR in the two villages was higher at T2 compared to T1 and T3. The highest HBR peak occurred between 19:00–20:00 h in Ambohitromby and between 21:00–22:00 h in Miarinarivo. *An. funestus* and *An. mascarensis* showed the lowest HBR peak in the two villages with the same biting pattern along the night.

#### Endophagy rate of malaria vectors

Endophagy rates, representing the proportion of mosquitoes collected indoors over the total number of mosquitoes collected indoors and outdoors by HLCs, are summarized in Table 4 for the four more abundant potential malaria vector species, according to the time-course of the survey and for each village. *Anopheles funestus* exhibited the highest endophagy rate in both villages (58.00 ± 4.94% in Ambohitromby and 53.66 ± 5.51% in Miarinarivo). The lowest endophagy rate in Ambohitromby was exhibited by *An. coustani* (5.73 ± 1.39%), while it was *An. mascarensis* that exhibited the lowest endophagy rate in Miarinarivo (11.76 ± 7.81%). *Anopheles arabiensis* and *An. coustani*, known as zoo-anthropophilic species, exhibited higher endophagy rates in Miarinarivo (42.64 ± 4.35% and 29.24 ± 2.09%, respectively) compared to Ambohitromby (25.27 ± 2.59% and 5.73 ± 1.39%, respectively). Comparing the endophagy rate by mosquito species, village and time point revealed a number of associations. All *Anopheles* species, except for *An. funestus* which did not differ significantly between indoors and outdoors, were collected in higher numbers outdoors, while *Anopheles arabiensis* and *An. coustani* were collected in higher numbers at T2 and T3, respectively. From 17 PSCs, a total of 70 mosquitoes were collected resting indoors (Additional file 1: Table S4). Among those 42.10% were *An. funestus*, 31.57% *An. arabiensis*, 15.78% *An. coustani* and 10.52% *An. mascarensis* in Ambohitromby, while only *An. funestus* (96.15%) and *An. mascarensis* (3.84%) were collected resting indoors in Miarinarivo.

**Table 4:** Proportion (in %) of the malaria vectors collected biting indoor by HLCs (endophagic rate)

Species	Ambohitromby				Miarinarivo			
	T1	T2	T3	Average proportion ± SE	T1	T2	T3	Average proportion ± SE
*An. coustani*	16.00 (12/75)	2.38 (1/42)	1.85 (3/162)	5.73 ± 1.39	31.82 (21/66)	38.14 (37/97)	25.89 (80/309)	29.24 ± 2.09
*An. arabiensis*	29.69 (19/64)	23.67 (49/207)	30.00 (3/10)	25.27 ± 2.59	56.25 (9/16)	37.04 (30/81)	50.00 (16/32)	42.64 ± 4.35
*An. funestus*	41.18 (14/34)	50.00 (13/26)	77.50 (31/40)	58.00 ± 4.94	88.89 (8/9)	50.00 (7/14)	49.15 (29/59)	53.66 ± 5.51
*An. mascarensis*	16.67 (4/24)	7.69 (1/13)	0 (0/12)	10.20 ± 4.32	20.00 (1/5)	0 (0/2)	10.00 (1/10)	11.76 ± 7.81

*Notes*: Numbers in parenthesis represent the number of mosquitoes collected indoor over the total number of mosquitoes collected indoor and outdoor. *Anopheles gambiae* was not taken into account in this table due to its low number

*Abbreviation*: SE, standard error = sqrt (p(1 – p)/n)

### *Plasmodium* carriage in *Anopheles* mosquitoes and entomological inoculation rate

Among 1715 anopheline mosquitoes captured by HLCs (*n* = 1650) and PSCs (*n* = 65), 1550 were tested for the presence of *Plasmodium* sporozoites by TaqMan and SYBR Green assays. As described in the methods section, DNA was first extracted from the head-thorax of individual mosquitoes and its quality assessed by amplification of the S7 gene. Using an equal volume of gDNA from at most 6 mosquitoes of the same species, 261 pools were assembled and tested for the presence of *Plasmodium* DNA, using the *Plasmodium* TaqMan assay. A total of 23 pools were found positive for *Plasmodium* DNA. Deconvolution of each positive pool to individual mosquito revealed that 28 mosquitoes carried *Plasmodium* DNA in their head-thorax, all of which had been captured by HLCs only. However, the SYBR Green assay for *P. falciparum*/*P. vivax* species detection was conclusive only for 13 (out 28) mosquitoes carrying either *P. falciparum* or *P. vivax* parasite (Table 5). The *Plasmodium* species of the 15 remaining TaqMan *Plasmodium-*positive mosquitoes could not be identified possibly due to the less effective SYBR Green assay used. It nevertheless cannot be excluded that these mosquitoes were infected with *P. malariae* or *P. ovale* or even lemur parasites as the TaqMan assay was targeting a region of the *18S* gene highly conserved among *Plasmodium* species. Overall, 9 mosquitoes were positive for *P. falciparum* and 4 for *P. vivax.* These mosquitoes belong to three anopheline species: *An. funestus*; *An. arabiensis*; and *An. coustani* (with the latter species being the more frequently infected one) (Table 5). Based on the species-specific assay (SYBR Green), the sporozoite rate (SR) varied from 0 to 1.4% according to the *Anopheles* species. Including all *Anopheles* species and all mosquitoes captured by HLCs and PSCs the overall SR was 0.84%. Of note, *An. rufipes*, an anopheline species which is not known being a malaria vector in Madagascar, was found positive by the TaqMan *Plasmodium* assay (2/40). As there are increased reports on its role in malaria transmission in other countries [26–28], it might be worth to include this species for *Plasmodium* sporozoite carriage in future surveillance programs.

**Table 5:** *Plasmodium* carriage in *Anopheles* mosquitoes analysed in pools and individually

Species	Total screened	Pools analysed	Positive pools	Positive mosquitoes	Positive in species screening	*Plasmodium* species (*n*)	Sporozoite rate (SR) (%)
*An. coustani*	714	122	10	14	7	*Pf* (6); *Pv* (1)	*Pf* (0.84); *Pv* (0.14)
*An. gambiae* (*s.l.*)	374	60	8	9	3	*Pf* (1); *Pv* (2)	*Pf* (0.27); *Pv* (0.54)
*An. funestus*	212	36	3	3	3	*Pf* (2); *Pv* (1)	*Pf* (0.94); *Pv* (0.47)
*An. squamosus*	116	20	0	0	0	_	0
*An. mascarensis*	59	10	1	0	0	_	0
*An. rufipes*	40	7	1	2	0	_	0
*An. maculipalpis*	35	6	0	0	0	_	0
Total	1550	261	23	28	13	*Pf* (9); *Pv* (4)	*Pf* (0.58); *Pv* (0.26)

*Abbreviations*: n, number of samples positive to *Plasmodium* species-specific; *Pf*, *P. falciparum; Pv*, *P. vivax*

*Notes*: Mosquitoes analysed include those collected by both HLCs and PSCs

**Fig. 5:**
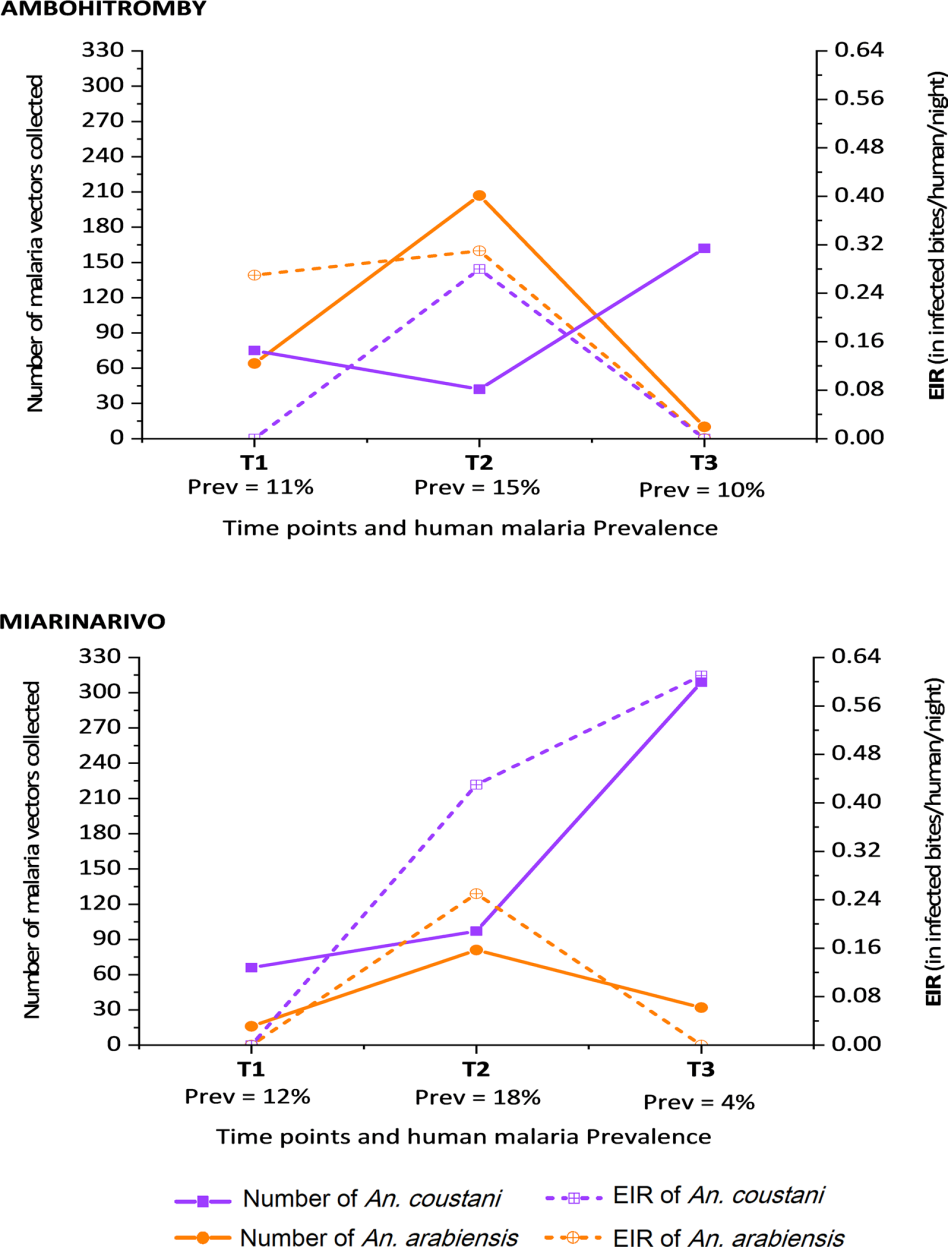
Variation of the density and EIR of *An. arabiensis* and *An. coustani* over time in Ambohitromby and Miarinarivo. Prev: human malaria prevalence

When looking at the entomological inoculation rate (EIR) as a proxy for malaria transmission, it appears that *An. arabiensis* and *An. coustani* contribute most to malaria transmission, but with striking differences between the two villages and over time (Table 6). Indeed, in Ambohitromby, *An. arabiensis* was the main vector at the beginning (T1) and the mid-term (T2) of transmission season, with EIRs of 0.27 and 0.31 ib/h/n respectively, followed by *An. coustani* at T2 (0.28 ib/h/n). By contrast, in Miarinarivo, *An. coustani* was the main vector at T2 and T3 (EIRs of 0.43 and 0.61 ib/h/n respectively), followed by *An. arabiensis* that played a vector role at T2 only (EIR of 0.25 ib/h/n). Plotting the number of *An. arabiensis* and *An. coustani*, and their respective EIR, show that the EIRs are not proportional to the number of captured mosquitoes and that this is evidenced in the two villages over the transmission season (Fig. 5). In Ambohitromby, *An. arabiensis *and *An. coustani* showed comparable densities at T1 with different EIRs (0.27 and 0 ib/h/n respectively); while their density at T2 and T3 was greatly different, but the EIR was similar for both species. In Miarinarivo, despite similar density of *An. arabiensis* and *An. coustani* at T2, *An. coustani *contributed most to malaria transmission and maintained this role at T3 with increased EIR possibly associated to its higher density.

**Table 6:** Entomological indices of malaria vectors at the three time points in the two villages

Species	Ambohitromby						Miarinarivo					
	Time point	HBR	*n*	(+)	SR (%)	EIR	Time point	HBR	*n*	(+)	SR (%)	EIR
*Anopheles coustani*	T1	6.25	68	0	0	0	T1	5.50	65	0	0	0
	T2	3.50	25	2	8.00	0.28	T2	8.08	95	5	5.26	0.43
	T3	13.50	159	0	0	0	T3	25.75	297	7	2.36	0.61
	Total	7.75	252	2	0.40	0.03	Total	13.11	457	12	2.63	0.34
*Anopheles arabiensis*	T1	5.33	58	3	5.08	0.27	T1	1.33	16	0	0	0
	T2	17.25	166	3	1.78	0.31	T2	6.75	81	3	3.70	0.25
	T3	0.83	10	0	0	0	T3	2.67	32	0	0	0
	Total	7.81	234	6	2.52	0.20	Total	3.58	129	3	2.34	0.08
*Anopheles funestus*	T1	2.83	35	1	2.86	0.08	T1	0.75	14	0	0	0
	T2	2.17	28	0	0	0	T2	1.17	16	1	6.25	0.07
	T3	3.33	42	0	0	0	T3	4.92	77	1	1.30	0.06
	Total	2.78	105	1	0.95	0.03	Total	2.38	107	2	1.87	0.04
All 3 species	T1	14.42	163	4	2.45	0.35	T1	7.58	94	0	0	0
	T2	22.92	222	5	2.25	0.52	T2	16.00	192	9	4.69	0.75
	T3	17.67	215	0	0	0	T3	33.33	406	8	1.97	0.66
	Total	18.33	600	9	1.50	0.27	Total	18.97	692	17	2.46	0.47

*Abbreviations*: HBR, human-biting rate (the number of collected mosquitoes per number of human-night (12 at each time point in each village)). The HBR is expressed in bite/human/night (b/h/n); n, the number of anopheline mosquitoes collected by HLCs and analysed by real time PCR. The DNA was extracted from individual head-thorax; (+) corresponds to the number of *Plasmodium* positive samples confirmed by the TaqMan *18S*; SR, sporozoite rate (the number of positive samples divided by the number of analysed samples (*n*), in %); EIR, entomological inoculation rate (EIR = HBR × SR). It is expressed as infective bite/human/night (ib/h/n)

## Discussion

The objective of this study, conducted in two neighbouring villages in a region of Madagascar where malaria is still a high public health problem, was to estimate the level of malaria transmission and to identify the mosquito vector species involved. Indeed, in that region, no such study had ever been conducted despite the high number of patients diagnosed with malaria at the local health centres. To our knowledge, the only study carried out at the same site, just provided entomological data and goes back to 1992 [29].

### Similarity in human malaria prevalence in the two villages

Parasitological data in the asymptomatic villagers revealed malaria infection cases mainly due to *P. falciparum*, in addition to low levels of *P. vivax* and *P. malariae*. The overall malaria prevalence throughout the study period was 11.9% as determined by PCR, and 4.8% by microscopy. These values are similar to the ones reported in the Tsiroanomandidy study performed in March 2014 [13]. Like the Tsiroanomandidy study, the results of this study highlight the high prevalence of sub-microscopic *Plasmodium* carriage which represents 50–75% of the investigated cases (negative by microscopy, positive by PCR).

**Fig. 6:**
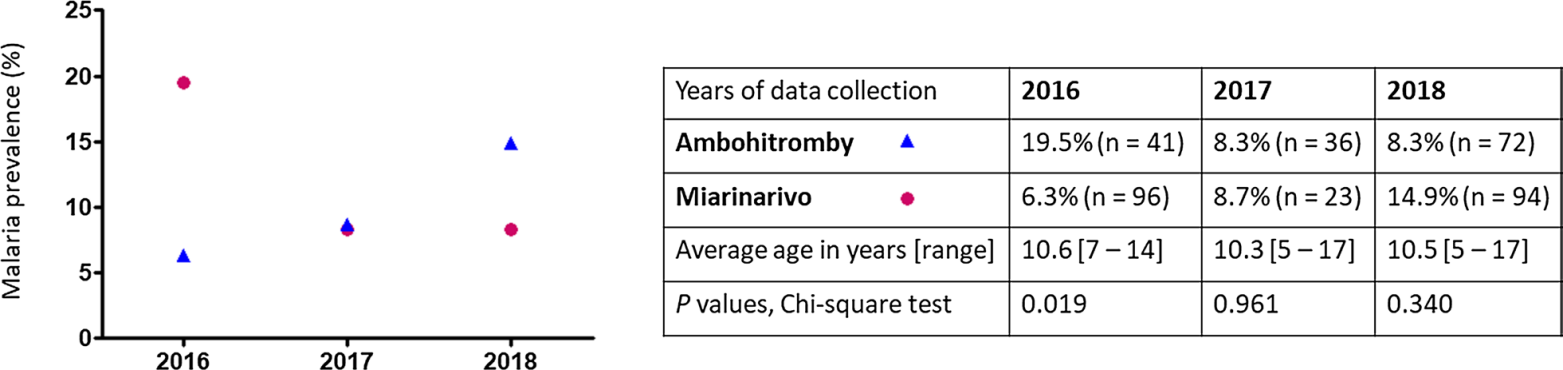
Malaria prevalence in Ambohitromby and Miarinarivo in 2016, 2017 and 2018. Data were collected from asymptomatic school-aged children tested with RDT in March each year (at T2). *P* values < 0.05 are significant; n: sample size

Data from this study show a similar malaria prevalence in the two villages. This is in sharp contrast to the significant difference in prevalence that was observed in 2016, between the school-age children of Ambohitromby and Miarinarivo (our unpublished data). Indeed, in 2016 a significant difference (*P* = 0.019, Chi-square test) in malaria RDT prevalence was observed between Ambohitromby (19.5%, *n* = 41) and Miarinarivo (6.3%, *n* = 96), but not in 2017 (this study) nor 2018 (Bourgouin et. al. unpublished data, Fig. 6). Such variation in malaria prevalence across years in the two villages, might result from better mosquito net coverage of the populations or climatic and ecological changes impacting *Anopheles* density [30, 31]. However, no mosquito net distribution was done between 2016 and 2018 in Andriba. Therefore, it might be possible that, in Ambohitromby 2016, the local conditions facilitated the development of an increased number of mosquito breeding sites leading to increased *Anopheles* vector population size and subsequent increased transmission. These results reflect the dynamic in malaria transmission in these two villages and the need to adapt locally vector control strategies.

### Variation in the number of malaria vector species is associated with time point and village

Entomological data from HLCs and PSCs, showed that *Anopheles* species diversity was similar between the two villages surveyed (Tables 3, 4). However, the analysis of total *Anopheles* caught by HLCs across the malaria transmission season revealed significant associations with time point and an interaction with village, especially for *An. arabiensis* and *An. coustani*. *Anopheles arabiensis* was the species with the highest HBR at the mid-term of the transmission season (February) in Ambohitromby, while *An. coustani* was the one with the highest HBR at the onset (December) and at the late-term (April) in both villages. This change in HBR over the malaria transmission season could be explained by changes in the ecological environment, precisely the rice fields which constitute the main *Anopheles* larval habitats in the two villages. Indeed, the growing phases of the rice determine important changes in the characteristics of the *Anopheles* breeding sites [32]. Fields with rice in the early stages of growth and with young short plants, offer sunny breeding sites favourable for the development of *An. gambiae* (*s.l*.) larvae. As the rice plants grow, they shade the water of the rice fields which become less favourable for *An. gambiae* (*s.l*.) larvae, giving way to *An. coustani* larvae that prefer shaded breeding sites. This transition from *An. gambiae* (*s.l*.) to *An. coustani* in breeding sites by increasing vegetation cover, was also demonstrated for borrow pits in Ethiopia [33].

Through the analysis of the *Anopheles* vector feeding behaviour, only *An. funestus* showed a strong tendency for both biting and resting indoor, not departing from its known behaviour in Andriba [29] and in other African regions including Madagascar [5, 17, 34–37]. Both *An. arabiensis* and *An. coustani* exhibited a significant outdoor biting preference in each village towards increased endophagy in Miarinarivo (Table 4). This observation is suggestive of the presence of different populations for both vector species in each village. However, it cannot be excluded though that the local environment such as distance between houses and breeding sites, their number and size, and the structure of the villages itself contribute to the observed differences in *An. arabiensis* and *An. coustani* endophagy between the two villages. The absence of indoor resting *An. arabiensis* and *An. coustani* in Miarinarivo despite their abundance, might also advocate for the presence of different populations for both species in each village.

### Different contribution of *Anopheles* species to malaria transmission between the two villages

Among 1550 mosquitoes tested, 28 were found positive for bearing *Plasmodium* sporozoites by TaqMan assay. The SYBR Green PCR assay allowed to identify either *P. falciparum* or *P. vivax* in 13 out of those 28 mosquito samples. Given the fact that the prevalence of *P. malariae* and *P. ovale* is very low in the studied community, this result suggests that the SYBR Green assay had a poor performance under our experimental condition, possibly linked to low sporozoite loads in the mosquito samples. As a consequence, it is difficult to discuss species specific rates (SR and EIR). All mosquitoes positive for *Plasmodium* spp were collected by HLCs, with 20/28 collected outdoors. Among the 28 *Plasmodium*-positive mosquitoes, 14 were *An. coustani*, 9 *An. arabiensis*, 3 *An. funestus* and 2 *An. rufipes*. Whereas *An. funestus* and *An. arabiensis* are well known malaria vectors in Madagascar, the contribution of *An. coustani* to malaria transmission has been suspected on several occasions due to its high density and propensity to anthropophily [38]. It was only recently that some *An. coustani* samples were detected CSP-positive by ELISA [9]. Data from this study, using a robust TaqMan assay, clearly demonstrated the vector role of *An. coustani* in malaria transmission in Andriba. *Anopheles coustani* is also known to be a malaria vector in continental Africa: in Cameroon [39]; Zambia [40, 41]; and Kenya [42]. This work also revealed that two out of 40 *An. rufipes* analysed by the TaqMan assay were found possibly carrying *Plasmodium* sporozoites. To date, *An. rufipes* has never been reported naturally infected with *Plasmodium* in Madagascar. In continental Africa, it was found naturally infected with *P. falciparum* in Burkina Faso [26, 43] and more recently in Cameroon [28]. Lastly, none of the *An. mascarensis* samples (*n* = 59) were found positive for *Plasmodium* although it is known as a malaria vector in other Malagasy areas [8, 10, 44, 45].

Surprisingly, this study revealed that *An. coustani*, was mainly responsible for malaria transmission in Miarinarivo, despite the presence of *An. funestus* and *An. arabiensis*. In that village, people were exposed to 61.2 ib/h infected bites per human (ib/h) from *An. coustani* during the study period (6 months), compared to only 5.4 ib/h in Ambohitromby, despite its relatively high abundance in this latter village. *Plasmodium* infected *An. coustani* (*n* = 12) were captured equally outdoor and indoor in Miarinarivo. By contrast, *An. arabiensis* was mainly responsible for malaria transmission in Ambohitromby, with 36 ib/h during the same study period, while it was responsible for 14.4 ib/h in Miarinarivo. The majority of infected *An. arabiensis* (9/10) were found outdoors. *Anopheles funestus* contributed to a minor extent to malaria transmission in both villages, being responsible for 5.4 and 7.2 ib/h during the whole survey in Ambohitromby and Miarinarivo respectively. Infected *An. funestus* (*n* = 3) were captured either indoors or outdoors. Overall, these results are similar to those observed in a study conducted in the Taveta district in Kenya, where malaria transmission, due to *An. coustani*, *An. arabiensis* and *An. funestus* occurred both indoors and outdoors [42]. The different contribution of malaria vector species might result from a different layout of the houses in each village and their distance from the rice fields as previously argued [45]. Indeed, the satellite view of the two villages shows that the houses in Miarinarivo are more numerous and very close to each other compared to houses in Ambohitromby, and that Miarinarivo is surrounded by more and closer rice fields, which is particularly favourable to the large number of *An. coustani* recorded in Miarinarivo.

In summary, the results of this study show that in neighbouring villages with a similar malaria prevalence in the human population, malaria transmission was driven by two different mosquito species and notably involved *An. coustani* as the major vector in Miarinarivo. Detailed analysis of the EIR over time (Table 6) shows that most malaria transmission occurred at the beginning and middle of the malaria transmission in Ambohitromby due to *An. arabiensis*, while occurring at the mid-course and vanishing of the malaria transmission season in Miarinarivo, due to *An. coustani*. Overall, the population in Ambohitromby was expected to receive 48.6 ib/h over the malaria season (November-April) compared to 84.6 ib/h in Miarinarivo.

## Conclusions

Overall, this study demonstrates the variability of vector biology dynamics between two neighbouring villages with similar ecological settings. This is the first time that *An. coustani* has been clearly demonstrated as playing a major contribution in malaria transmission in an area of Madagascar, despite the presence of *An. arabiensis* and *An. funestus* known as major malaria vectors in the country. This finding was quite surprising as *An. coustani* is being known as a zoophilic and exophilic species in most areas of Madagascar where it was found. The results of this study can be used to better describe the epidemiology and transmission of malaria in Madagascar and to provide relevant information as guidance for adapted malaria vector control strategies. In an epidemiological context such as Madagascar, marked by the presence of both *P. falciparum* and *P. vivax* in combination with presence of several vector species, understanding the vector-specific contributions to the transmission of these two main *Plasmodium* species constitutes a challenge for malaria elimination.

## Supplementary information

**Additional file 1: Table S1.** Sequences of the primers and TaqMan probes used for the morphological identification of *An. gambiae*/*An. arabiensis* and for *Plasmodium* detection in *Anopheles* mosquitoes. **Table S2.** Human population that participated in the study categorised by age group and sex. **Table S3.** Mosquitoes collected by HLCs inside and outside houses, in Ambohitromby and Miarinarivo at the three time points. **Table S4.** Number of mosquitoes collected resting indoor by PSC.

## Data Availability

All data generated or analysed during this study are included in this published article and its additional files.

## Abbreviations

ACT: artemisinin-based combination therapy
CSP: circumsporozoite protein
DNA: deoxyribonucleic acid
EIR: entomological inoculation rate
ELISA: enzyme-linked immunosorbent assay
GLMM: generalized linear mixed model
GPS: global positioning system
HLC: human landing catch
HN: human-night
ib/h/n: infective bite per human per night
PBS: phosphate-buffered saline
PCR: polymerase chain reaction
PSC: pyrethrum spray catch
RDT: rapid diagnostic test
RNA: ribonucleic acid
SE: standard error
SR: sporozoite rate
