# Supplementary material for: Differential contribution of Anopheles coustani and Anopheles arabiensis to the transmission of Plasmodium falciparum and Plasmodium vivax in two neighbouring villages of Madagascar
Source: Parasit Vectors. 2020 Aug 26;13:430. doi: 10.1186/s13071-020-04282-0 (PMC7447585; doi:10.1186/s13071-020-04282-0)
Supplement: Supplementary file 1 — Additional file 1: Table S1. Sequences of the primers and TaqMan probes used for the morphological identification of An. gambiae/An. arabiensis and for Plasmodium detection in Anopheles mosquitoes. Table S2. Human population that participated in the study categorised by age group and sex. Table S3. Mosquitoes collected by HLCs inside and outside houses, in Ambohitromby and Miarinarivo at the three time points. Table S4. Number of mosquitoes collected resting indoor by PSC. [file 13071_2020_4282_MOESM1_ESM.docx]

**Additional file 1: Table S1.** Sequences of the primers and TaqMan probes used for the morphological identification of *An. gambiae*/*An. arabiensis* and for *Plasmodium* detection in *Anopheles* mosquitoes.

| **Species** | **Primer/Probe** | **Sequence** | **Reference** |
| --- | --- | --- | --- |
| *An. gambiae* (*s.l*.) | UNI-F | 5'-GTGAAGCTTGGTGCGTGCT-3' | Walker *et al.,* 2007 |
| *An. gambiae* (*s.l.*) | UNI-R | 5'-GCACGCCGACAAGCTCA-3' |  |
| *An. gambiae* | Probe | 5'-VIC- CGGTATGGAGCGGGACACGTA-MGB |  |
| *An. arabiensis* | Probe | 5'-6FAM-TAGGATGGAGAAGGACACTTA-MGB |  |
| *Plasmodium* *spp.* | Plasmo1-F | 5'-GTT AAG GGA GTG AAG ACG ATC AGA | Modified from Rougemont *et al*., 2004 |
|  | Plasmo2-R | 5'-AAC CCA AAG ACT TTG ATT TCT CAT AA |  |
|  | Plasmoprobe | 5'-NED-TCGTAATCTTAACCATAAAC-MGB |  |
| *P. falciparum*-Cytb | Cytb F | 5’-ATGGATATCTGGATTGATTTTATTTATGA | Canier *et al.*, 2013 |
|  | Cytb R | 5’- TCCTCCACATATCCAAATTACTGC |  |
| *P. vivax*-Cytb | Cytb F | 5’- TGCTACAGGTGCATCTCTTGTATTC |  |
|  | Cytb R | 5’- ATTTGTCCCCAAGGTAAAACG |  |

**Additional file 1: Table S2.** Human population that participated in the study categorised by age group and sex

|  | |  | **Ambohitromby** | **Miarinarivo** | **Total** |
| --- | --- | --- | --- | --- | --- |
|  | Sample size | | 218 | 162 | 380 |
| Age group | <5 | | 34 | 28 | 62 (16.32%) |
|  | [5-10[ | | 43 | 24 | 67 (17.63%) |
|  | [10-15[ | | 46 | 23 | 69 (18.16%) |
|  | >15 | | 95 | 87 | 182 (47.89%) |
| Sex | Male | | 97 | 75 | 172 (45.26%) |
|  | Female | | 121 | 87 | 208 (54.73%) |

The numbers in parenthesis are the proportions.

**Additional file 1: Table S3.** Mosquitoes collected by HLCs inside and outside houses, in Ambohitromby and Miarinarivo at the three time points.

|  | **Ambohitromby** | | | | | | | |  | **Miarinarivo** | | | | | | | | **Total** |
| --- | --- | --- | --- | --- | --- | --- | --- | --- | --- | --- | --- | --- | --- | --- | --- | --- | --- | --- |
|  | T1 | | T2 | | T3 | | Total | |  | T1 | | T2 | | T3 | | Total | |  |
| Mosquito species | Ind | Out | Ind | Out | Ind | Out | Ind | Out |  | Ind | Out | Ind | Out | Ind | Out | Ind | Out |  |
| *Anopheles coustani* | 12 | 63 | 1 | 41 | 3 | 159 | 16 | 263 |  | 21 | 45 | 37 | 60 | 80 | 229 | 138 | 334 | 751 |
| *Anopheles arabiensis* | 19 | 45 | 49 | 158 | 3 | 7 | 71 | 210 |  | 9 | 7 | 30 | 51 | 16 | 16 | 55 | 74 | 410 |
| *Anopheles funestus* | 14 | 20 | 13 | 13 | 31 | 9 | 58 | 42 |  | 8 | 1 | 7 | 7 | 29 | 30 | 44 | 38 | 182 |
| *Anopheles squamosus/cydippis* | 1 | 12 | 0 | 43 | 3 | 26 | 4 | 81 |  | 0 | 8 | 19 | 21 | 4 | 11 | 23 | 40 | 148 |
| *Anopheles mascarensis* | 4 | 20 | 1 | 12 | 0 | 12 | 5 | 44 |  | 1 | 4 | 0 | 2 | 1 | 9 | 2 | 15 | 66 |
| *Anopheles rufipes* | 2 | 7 | 2 | 8 | 0 | 0 | 4 | 15 |  | 2 | 3 | 6 | 5 | 4 | 5 | 12 | 13 | 44 |
| *Anopheles maculipalpis* | 0 | 7 | 0 | 9 | 0 | 0 | 0 | 16 |  | 5 | 5 | 1 | 8 | 2 | 3 | 8 | 16 | 40 |
| *Anopheles gambiae* | 0 | 0 | 1 | 3 | 0 | 0 | 1 | 3 |  | 1 | 0 | 2 | 0 | 1 | 0 | 4 | 0 | 8 |
| *Anopheles pretoriensis* | 0 | 0 | 0 | 0 | 0 | 0 | 0 | 0 |  | 0 | 0 | 0 | 0 | 1 | 0 | 1 | 0 | 1 |
| *Culex antennatus* | 3 | 27 | 38 | 220 | 0 | 4 | 41 | 251 |  | 26 | 23 | 35 | 64 | 4 | 21 | 65 | 108 | 465 |
| *Culex quinquefasciatus* | 1 | 4 | 40 | 63 | 0 | 0 | 41 | 67 |  | 4 | 11 | 8 | 15 | 1 | 18 | 13 | 44 | 165 |
| *Culex giganteus* | 0 | 0 | 0 | 0 | 0 | 0 | 0 | 0 |  | 1 | 1 | 2 | 2 | 0 | 2 | 3 | 5 | 8 |
| *Culex univittatus* | 0 | 4 | 0 | 0 | 0 | 0 | 0 | 4 |  | 0 | 0 | 0 | 0 | 0 | 0 | 0 | 0 | 4 |
| *Culex decens* | 0 | 1 | 0 | 0 | 0 | 0 | 0 | 1 |  | 1 | 1 | 0 | 0 | 0 | 0 | 1 | 1 | 3 |
| *Culex bitaeniorhyncus* | 0 | 0 | 0 | 0 | 0 | 0 | 0 | 0 |  | 0 | 1 | 0 | 1 | 0 | 0 | 0 | 2 | 2 |
| *Mansonia uniformis* | 1 | 7 | 2 | 7 | 1 | 2 | 4 | 16 |  | 2 | 3 | 4 | 21 | 4 | 30 | 10 | 54 | 84 |
| *Aedes tiptoni* | 0 | 1 | 0 | 0 | 0 | 0 | 0 | 1 |  | 2 | 6 | 0 | 0 | 1 | 0 | 3 | 6 | 10 |
| *Aedes skusea* | 0 | 5 | 0 | 0 | 0 | 0 | 0 | 5 |  | 1 | 2 | 0 | 0 | 0 | 0 | 1 | 2 | 8 |
| *Aedes albopictus* | 0 | 0 | 0 | 1 | 0 | 0 | 0 | 1 |  | 1 | 0 | 1 | 0 | 0 | 0 | 2 | 0 | 3 |
| *Aedes vittatus* | 0 | 1 | 0 | 1 | 0 | 0 | 0 | 2 |  | 0 | 0 | 0 | 0 | 0 | 0 | 0 | 0 | 2 |
| *Aedes circumlateolus* | 0 | 0 | 0 | 0 | 0 | 0 | 0 | 0 |  | 0 | 0 | 0 | 0 | 0 | 1 | 0 | 1 | 1 |
| *Coquillettidia grandidieri* | 0 | 0 | 0 | 0 | 0 | 0 | 0 | 0 |  | 0 | 0 | 0 | 0 | 0 | 2 | 0 | 2 | 2 |
| Total | 57 | 224 | 146 | 576 | 41 | 219 | 245 | 1022 |  | 84 | 121 | 150 | 257 | 147 | 377 | 385 | 755 | 2407 |

Ind: Indoor; Out: Outdoor

**Additional file 1: Table S4.** Number of mosquitoes collected resting indoor by PSC.

|  | **Ambohitromby** | | | | **Miarinarivo** | | | | **Total** |
| --- | --- | --- | --- | --- | --- | --- | --- | --- | --- |
| Species | T1 | T2 | T3^a^ | Total | T1 | T2^b^ | T3 | Total |  |
| *An. coustani* | 2 | 0 | 4 | 6  (15.78%) | 0 | 0 | 0 | 0 | 6  (9.37%) |
| *An. arabiensis* | 9 | 3 | 0 | 12  (31.57%) | 0 | 0 | 0 | 0 | 12  (18.75%) |
| *An. funestus* | 10 | 4 | 2 | 16  (42.10%) | 5 | 2 | 18 | 25  (96.15%) | 41  (64.06%) |
| *An. mascarensis* | 3 | 0 | 1 | 4  (10.52%) | 1 | 0 | 0 | 1  (3.84%) | 5  (7.81%) |
| *An. rufipes* | 1 | 0 | 0 | 1 | 0 | 0 | 0 | 0 | 1 |
| *Cx antennatus* | 0 | 0 | 0 | 0 | 0 | 2 | 0 | 2 | 2 |
| *Cx quinquefasciatus* | 0 | 3 | 0 | 3 | 0 | 0 | 0 | 0 | 3 |
| Total | 25 | 10 | 7 | 42 | 6 | 4 | 18 | 28 | 70 |

**^a^** Captures were performed for 2 days only. **^b^** Captures were performed in 2 houses only. Values in brackets represent the relative abundance (endophilic rate) to known malaria vectors collected resting indoor (*An. arabiensis, An. funestus, An. mascarensis* and *An. coustani*), for a total of 38 mosquitoes in Ambohitromby and 26 in Miarinarivo. 3 PSCs were performed at each time point per village, except in Ambohitromby at T3 where only 2 could be performed; this led to a total of 17 PSCs, 8 in Ambohitromby and 9 in Miarinarivo.
